# A rare presentation of Sinonasal Undifferentiated Carcinoma with brain metastasis and para-aortic mass

**DOI:** 10.4322/acr.2020.222

**Published:** 2020-11-20

**Authors:** Sujata Sarangi, Sudeep Khera, Vikarn Vishwajeet, Vikas Meshram, Puneet Setia, Abhishek Malik

**Affiliations:** 1 All India Institute of Medical Sciences, Department of Pathology & Laboratory Medicine, Jodhpur, Rajasthan; 2 All India Institute of Medical Sciences, Department of Forensic Medicine and Toxicology, Jodhpur, Rajasthan

**Keywords:** Maxillary Sinus, Sinonasal undifferentiated Carcinoma, Metastasis

## Abstract

Sinonasal Undifferentiated carcinoma (SNUC) comprises 3% of the head and neck tumors, including metastatic neoplasms. Herein we report the case of a 60-year-old male who was brought dead to our institute with previous records of a contrast-enhanced CT scan of the brain and MRI with evidence of tumor in the maxillary sinus with intracranial extensions. The histopathological examination of the mass in the maxillary sinus proved to be SNUC with metastases to the brain, lungs, and around the aorta. These tumors are undifferentiated and are distinct from other poorly differentiated tumors in deriving their origin from the Schneiderian epithelium. The aggressive nature of the tumor renders the prognosis quite dismal. SNUCs need to be early recognized and distinguished from other poorly differentiated carcinomas with the help of immunohistochemistry.

## INTRODUCTION

Sinonasal Undifferentiated carcinoma (SNUC) is a rare malignancy of the head and neck region comprising 3% of all tumors.^1^ First described by Frierson et al.^2^ in 1986, these tumors are distinct from other head and neck malignancies as it is hypothesized to arise from the Schneiderian epithelium or from the nasal ectoderm of the paranasal sinuses. SNUC is a high-grade epithelial neoplasm with or without neuroendocrine differentiation but without squamous or glandular differentiation.^2^ It is a highly aggressive tumor that has been seen extending beyond the sinonasal tract, to the skull base, orbit, and intracranium.^1^ ^,^ ^3^ SNUC usually involve the elderly with a slight male predominance (2-3:1) and presents a high rate of locoregional recurrences and metastases.^4^ ^,^ ^5^

## CASE REPORT

A 60-year-old male was brought dead to our institute with the history of cough and breathlessness. The patient was seeking treatment for his illness, as documented by accompanying relatives. Further detailed clinical history was not available, as the case was brought for medicolegal autopsy by police personnel due to sudden death.

The patient’s available records included a contrast-enhanced CT scan of the head taken 3 months back, showing two hyperdense, and non-enhancing brain parenchymal lesions in the left frontal and occipital lobes, measuring 11×10 mm and 10×10 mm, suggestive of neoplastic origin. The brain MRI showed multiple heterogeneous signal lesions with peripheral enhancement and perilesional edema involving the right capsular region, left frontal lobe, left occipital lobe, and the right cerebellum. The lesions were considered as possible metastasis. The paranasal sinus showed a large heterogeneous signal mass lesion involving the right maxillary sinus with an invasion of the right zygomatic bone, arch, and right sphenoidal vein with intracranial extension, causing compression over the right anterior temporal lobe

An autopsy was performed.

## AUTOPSY FINDINGS

The external examination revealed a body of a well-nourished man with a swelling over the zygomatic and maxillary area. The examination of the skull revealed a pinkish-white soft mass over the anterior and middle cranial fossa. The mass involved the right sphenoidal bone and extended into the right maxillary sinus and zygomatic bone. The left lung weighed 672 g, and the right lung weighed 780 g (mRR:450 g and 375 g, respectively). On cut section, reddish-brown fluid oozed out. There was no evidence of thrombus or consolidation. The thoracic cavity was unremarkable.

In the mediastinum, a hard mass was found surrounding the aortic arch. The Pathology Department received a soft mass from the maxillary sinus, the whole brain, the whole dissected heart, a fragment of the abdominal aorta, part of the aorta with the hard mediastinal mass, both lungs, two pieces of the kidneys, pieces of the pancreas, spleen, and liver.

**Figure 1:**
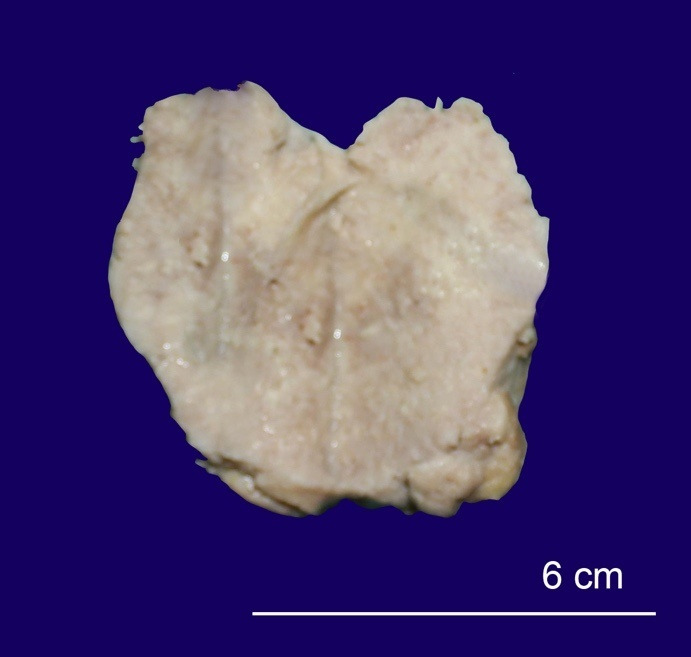
Gross view of the cut surface of the maxillary sinus soft mass.

The soft mass from the maxillary sinus measured 5.7×3.5×2.5 cm and weighed 27.4 gm. The cut section was grayish-white (Figure 1).

**Figure 2:**
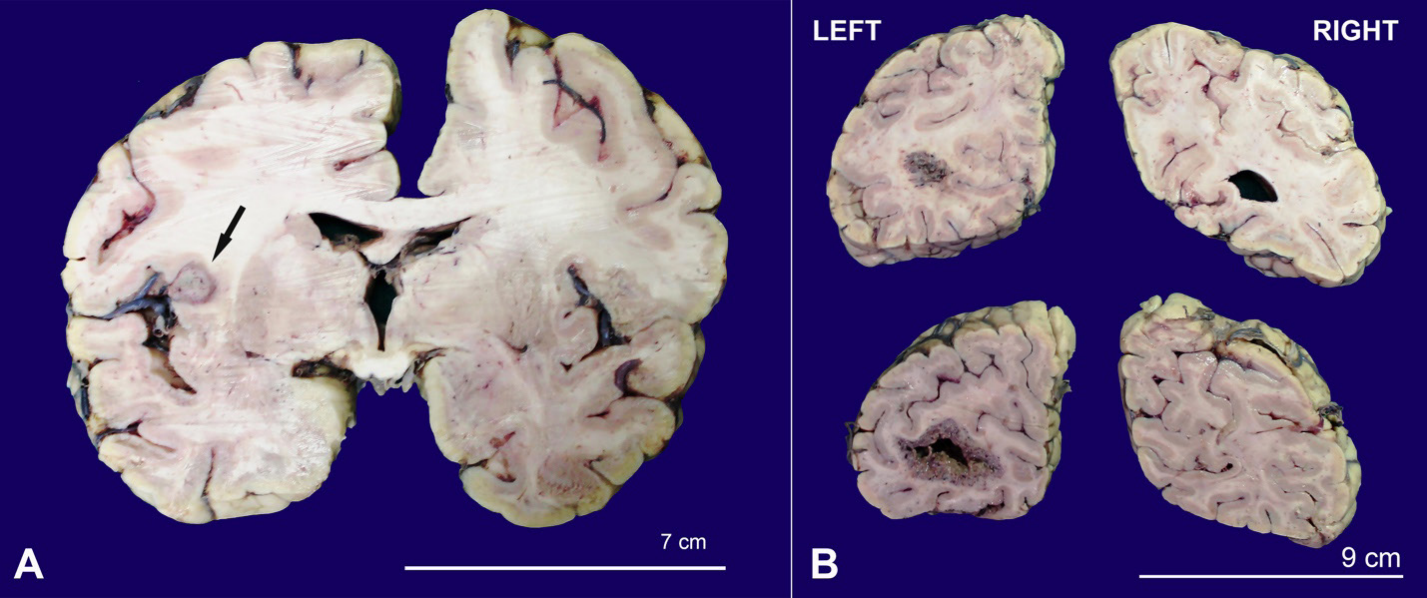
**A –** Gross view of the cerebrum showing a metastatic nodule (arrow); **B –** Gross view of the occipital lobe with evidence of metastasis.

The brain weighed 1308 g after autopsy and 1364.5 g post-fixation (reference range:1179-1621 g). The external surface showed normal gyri and sulci. No herniation was seen. On the coronal slicing, a lesion, measuring 1×0.8 cm, was seen in the insular cortex at the junction of the right cerebral hemisphere’s grey and white matters. On the left hemisphere, a lesion measuring 1×0.8×0.8 cm was seen at the grey and white matter junction (Figure 2A). The lesion was soft, grey-white with perilesional edema. There was a large lesion in the left occipital area, measuring 3×1.5×4.5 cm (Figure 2B). On slicing the cerebellum, there was a large and well-defined lesion measuring 3.3×1.6×2.5 cm involving the left cerebellum. The right cerebellum showed a small lesion measuring 0.8×0.5 cm. Representative sections from these lesional areas were submitted to histological examination. The heart’s gross examination showed 60% of luminal occlusion of the left anterior descending artery at its origin. A sample of the abdominal aorta was received measuring 7.5 cm in length. The lumen showed multiple hardened atheromatous plaques.

**Figure 3:**
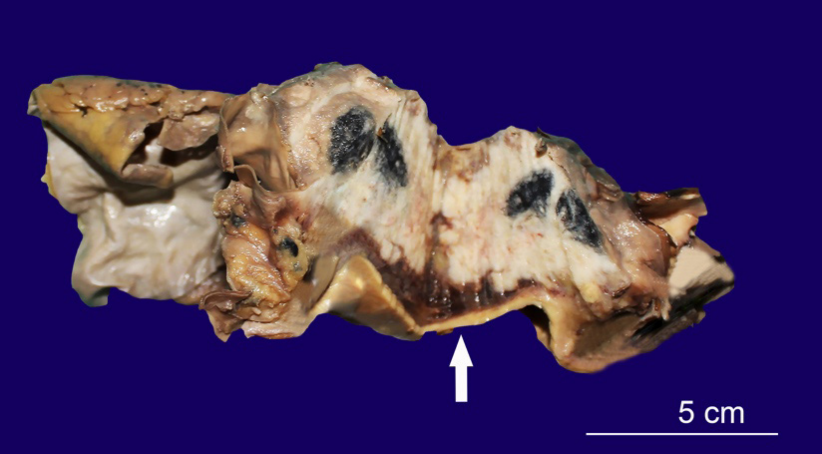
Gross view of the hard mass around the aorta. The white arrow points to the wall of the aorta.

The representative fragment of the aorta with an attached hard mass measured 10.1×6.7×41 cm. The aortic arch measured 4.5 cm in length (Figure 3).

The luminal diameter at one end was 2 cm, and at the other end was 1.5 cm. The whole hard mediastinal mass weighed 178.1 g. The cut section of the mass showed a whitish growth with central blackish discoloration abutting the wall of the arch of the aorta, which was thickened and showed atheromatous plaque.

Both lungs’ gross examination showed irregular and whitish nodules, which at the cut surface exhibited a central blackish discoloration measuring 2×2.5×4.2 cm and 4×3 cm, respectively.

The representative samples of the kidneys, pancreas, spleen, and liver did not show any gross evidence of tumor involvement.

Sections from the mass in the maxillary sinus showed a tumor arranged in the acinar and pseudo-alveolar pattern. The individual cells showed conspicuous to inconspicuous nucleoli with mild to moderate nuclear pleomorphism and abundant eosinophilic cytoplasm along with areas of necrosis. Increased mitotic count was observed. Occasional nuclear grooves and bizarre forms were identified. Numerous entrapped nerves were seen (Figure 44B). There were no areas of squamous, glandular, rhabdoid, or plasmacytoid differentiation.

**Figure 4:**
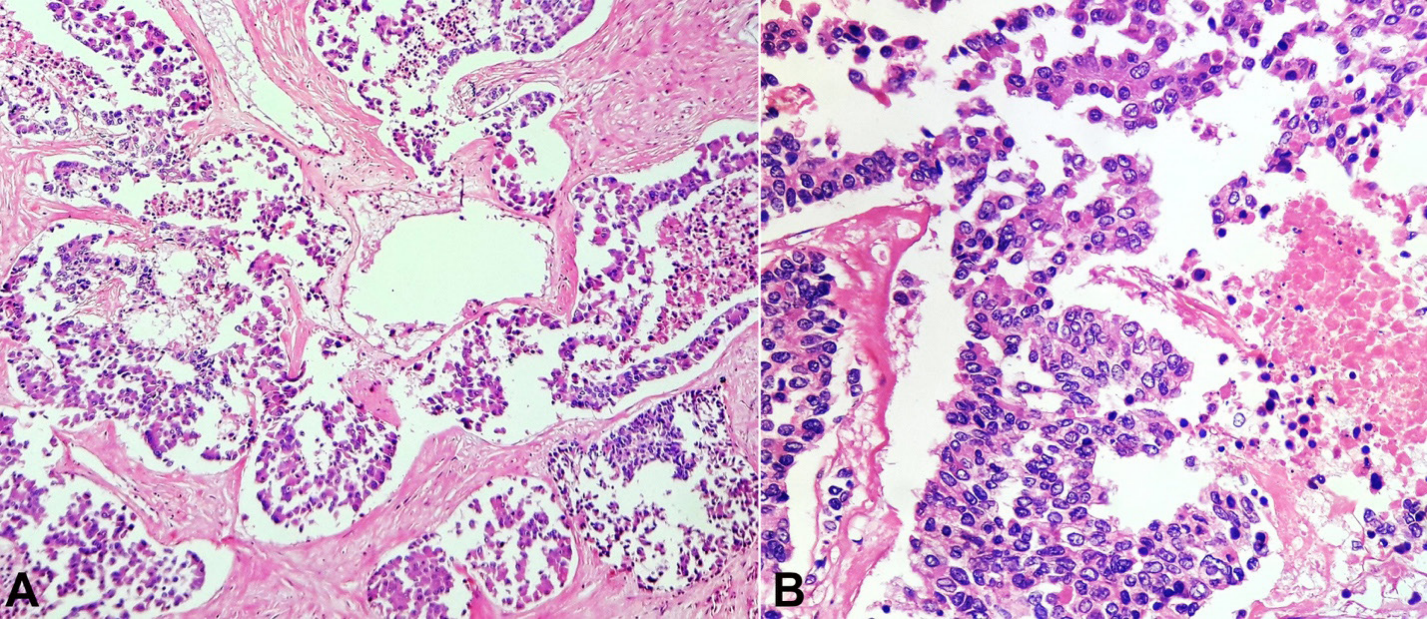
Photomicrographs of the maxillary sinus tumor. **A –** Undifferentiated Carcinoma with comedo necrosis (H&E,10X); **B –** individual cells show moderate to abundant eosinophilic cytoplasm and hyperchromatic to the vesicular nucleus(H&E,40X).

**Figure 5:**
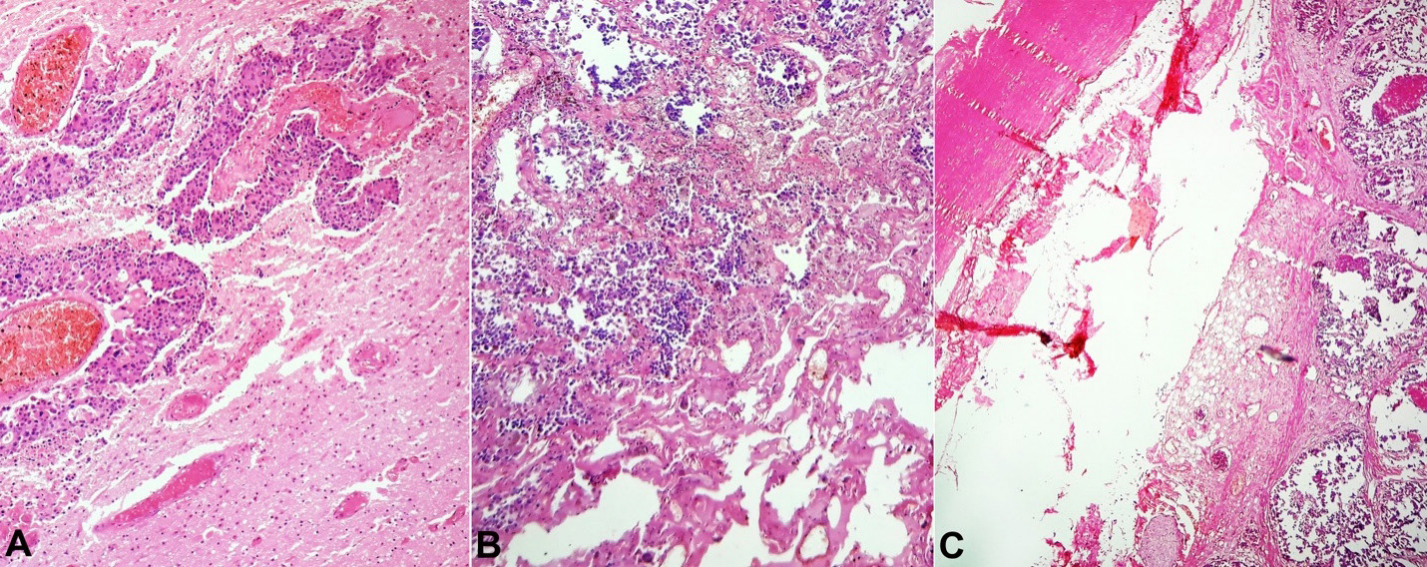
Photomicrographs of **A –** Brain; **B –** Lung and **C –** Aorta – showing metastases, (H&E,10X, 10X, and 4X respectively).

Sections from the cerebrum and cerebellum showed metastatic deposits (Figure 5A).

Sections from the heart showed atheromatous changes in the main coronary artery and left anterior descending artery with 50% occlusion at a distance of 3 cm from the origin. However, no myocardial infarction was noted, ruling out an ischemic myocardial disease as a possibility of the immediate cause of death. The wall of the abdominal aorta showed atheromatous plaque.

Sections from both lungs’ lesions (Figure 5B) and the hard mass showed metastases from the aforementioned tumor (Figure 5C) and an atheromatous plaque in the wall of the aorta. The remaining organs did not show evidence of metastases.

**Figure 6:**
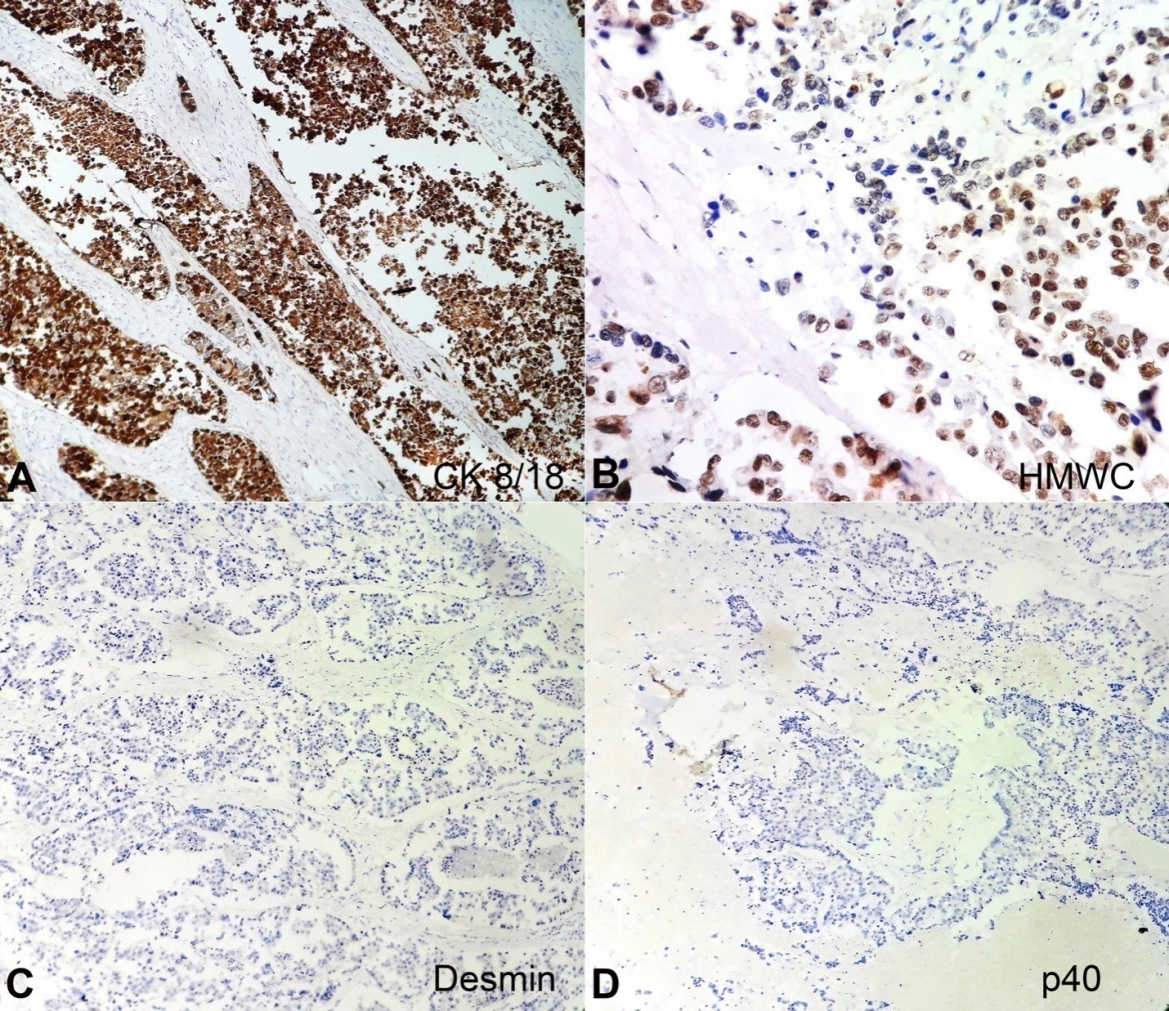
Photomicrographs of the tumor – **A** and **B –** positive reaction for CK 8/18 (10X); positive focal reaction for HMWC (40X); **C** and **D –** negative reaction for Desmin and p40 respectively (10X both).

**Figure 7:**
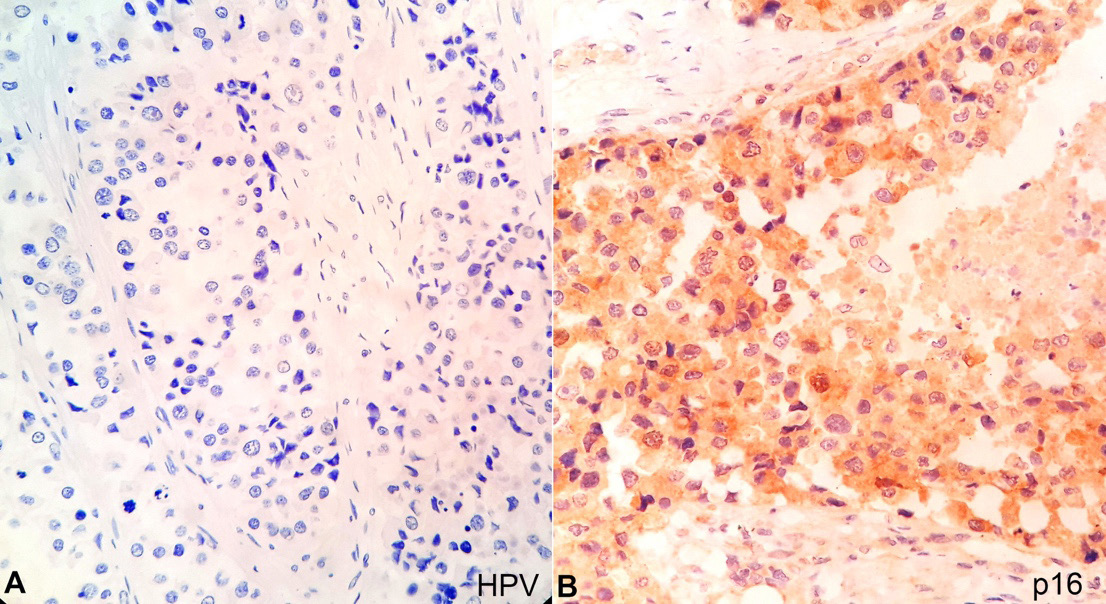
Photomicrographs of the tumor. **A –** negative reaction for Human Papilloma Virus (HPV)(40X); **B –** Focal positive reaction for p16 (40X).

**Figure 8:**
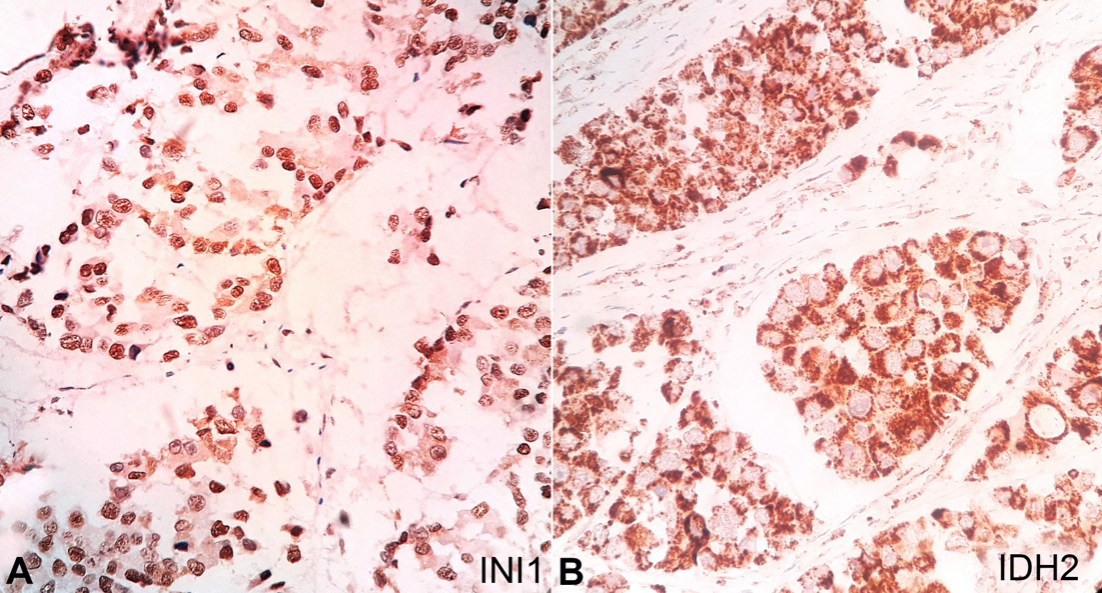
Photomicrographs of the tumor. **A –** positive reaction for INI 1 (40X); **B –** positive reaction for IDH 2 (40X).

The sections from the soft mass subjected to immunohistochemistry (IHC) reacted positively for CK 8/18 (Figure 6A) and high molecular weight cytokeratin (Figure 6B) and negatively for desmin and p40 (Figure 66D). The tumor was negative for vimentin, synaptophysin, Human Papilloma Virus (HPV, Figure 7A), Leucocyte Common Antigen, and HMB45but was focally positive for p16 (Figure 7B). The Ki67 proliferative index was 30%. The tumor showed retention of INI-1 ruling out SMARCB1-deficient tumors (Figure 8A). Additionally, it was positive for IDH-2 codon R172 mutant on IHC (Figure 8B).

The post mortem diagnosis was of Sinonasal Undifferentiated Carcinoma arising from maxillary sinus with metastasis to brain, soft tissue around the aorta, and bilateral lungs.

## DISCUSSION

Sinonasal Undifferentiated carcinoma (SNUC) is a rare and highly aggressive tumor with an uncertain histogenesis and etiology.^6^ In this setting, the associations with tobacco smoking and radiation have been raised.^7^

SNUCs are more commonly seen in males and are more prevalent in the elderly with the mean age ranging between55-60 years of age.^8^ Our case concurred for age and gender.

The most common symptoms are nasal obstruction, periorbital pain and swelling, proptosis, diplopia, and cranial nerve palsies.^1^ Due to the invasive nature of the tumor, the destruction of the adjacent structures is observed, like infiltration of the nasopharynx, and intracranial extension through the orbital plates, which are well demonstrated on CT or MRI. Our case is a classic example of the tumor's nature as the patient was brought dead with metastases to the brain, lungs, and soft tissue surrounding the aorta.

On light microscopy, the tumor is generally arranged in sheets, nests, and trabecular patterns with small to medium polygonal cells with moderate amphophilic cytoplasm, vesicular to hyperchromatic nucleus and inconspicuous to prominent nucleoli. Brisk mitotic activity is noted along with prominent areas of necrosis and angiolymphatic invasion, which were observed in our case.^8^ There were no areas of squamous, glandular, or rhabdoid differentiation in our tumor, ruling out the possibility of NUT-carcinoma or SMARCB1 deficient tumors.

The differential diagnosis of SNUCs includes (i) nasopharyngeal carcinoma; (ii) esthesioneuroblastoma; (iii) neuroendocrine carcinoma; (iv) rhabdomyosarcoma; (v) lymphoepithelioma; (vi) lymphoma; (vii) melanoma; and (viii) poorly differentiated adenoid cystic carcinoma. Therefore, immunohistochemistry is fundamental for the precise diagnosis.^1^SNUCs are immunoreactive for CK7, CK8/18, CK19, and epithelial membrane antigen (EMA) and negative for synaptophysin, chromogranin, vimentin, and CD45, these reactions are necessary to rule out all the other poorly differentiated malignancies of the head and neck region.^8^ ^,^ ^9^

Our case was immunoreactive for CK 8/18, high molecular weight cytokeratin and focally for p16and negative for vimentin, desmin, p40, synaptophysin, Human Papilloma Virus (HPV), Leucocyte Common Antigen, and HMB45, thus ruling out the possibility of a sarcoma, squamous cell carcinoma, and melanocytic tumour.^10^

Moreover, to rule out SNUC-like neoplasms, immunohistochemistry for INI-1 and IDH-2 was also performed. INI-1 was found retained ruling out the possibility of SMARCB1 deficient tumors, and IDH-2 mutant was positive reinforcing the diagnosis of SNUC. Hence, a diagnosis of SNUC was rendered. The differential diagnosis of SNUC-like tumors has been enumerated in the Table 1 below.

**Table 1:** Adapted from Agaimy et al.,^11^ and Weindorf et al.^12^

**Differential diagnosis of sinonasal tumors**	**Clinical features**	**Morphological** **features**	**Molecular features**
Sinonasal Undifferentiated Carcinoma (SNUC)	30-77 years; nasal cavity and maxillary sinus; Aggressive uncommon	Undifferentiated without any squamous, glandular or rhabdoid differentiation^7^	IDH2 codon R172 mutation^11^ ^,^ ^12^
NUT- carcinoma	26-48 years; Sinuses. Aggressive tumors	Undifferentiated with a squamous differentiation	NUTM1 gene re-arrangement^12^
Sinonasal adenocarcinoma	Aggressive tumors	Enteric and mucinous subtype	MAPK mutations and ETV6 gene rearrangements^12^
SMARCB1 deficient tumors	19-89 years; sinuses, mainly ethmoid. Aggressive tumors	Plasmacytoid and rhabdoid	loss of INI-1^12^
SMARCA4deficient carcinomas	20-67 years; mainly nasal cavity; Aggressive tumors	Poorly differentiated	SMARCA4 deficient^13^
Sinonasal squamous cell carcinoma	Most common	Keratinizing, non-keratinizing, adenosquamous basaloid.	HPV subtypes expression, KRAS, and EGFR mutations^12^

The treatment protocol advised in the available literature is radical curative resection followed by adjuvant chemo-radiation.^14^ ^,^ ^15^ However, given the disease’s aggressiveness, it has been noted that even extensive management protocols and a stringent follow up do not exempt these cases from progression and metastasis.

Unfortunately, in our case, the patient succumbed to the disease with widespread metastases due to a probable diagnosis delay. As we could not elicit a detailed clinical history, we cannot comment upon the disease’s course.

## CONCLUSION

The purpose of this case report is to emphasize the highly infiltrating nature of this tumor and the various implications it may have due to widespread metastasis if left untreated. Ignored cases might lead to the infiltration and failure of multiple organ systems to such an extent that the tumor itself may not remain the only cause of the patient’s suffering or death.
